# Evolution of variation in presence and absence of genes in bacterial pathways

**DOI:** 10.1186/1471-2148-12-55

**Published:** 2012-04-20

**Authors:** Andrew R Francis, Mark M Tanaka

**Affiliations:** 1 School of Computing, Engineering and Mathematics, University of Western Sydney, Penrith 2751, Locked Bag 1797, Australia; 2 School of Biotechnology & Biomolecular Sciences, University Of New South Wales, Kensington 2052, Australia

**Keywords:** Bacteria, Evolution, Pathway, Cryptic genetic variation, de Finetti, Mathematical model, Horizontal gene transfer, Reductive evolution, Genome evolution

## Abstract

**Background:**

Bacterial genomes exhibit a remarkable degree of variation in the presence and absence of genes, which probably extends to the level of individual pathways. This variation may be a consequence of the significant evolutionary role played by horizontal gene transfer, but might also be explained by the loss of genes through mutation. A challenge is to understand why there would be variation in gene presence within pathways if they confer a benefit only when complete.

**Results:**

Here, we develop a mathematical model to study how variation in pathway content is produced by horizontal transfer, gene loss and partial exposure of a population to a novel environment.

**Conclusions:**

We discuss the possibility that variation in gene presence acts as cryptic genetic variation on which selection acts when the appropriate environment occurs. We find that a high level of variation in gene presence can be readily explained by decay of the pathway through mutation when there is no longer exposure to the selective environment, or when selection becomes too weak to maintain the genes. In the context of pathway variation the role of horizontal gene transfer is probably the initial introduction of a complete novel pathway rather than in building up the variation in a genome without the pathway.

## Background

The analysis of bacterial genomes has revealed a remarkable degree of variation in the presence and absence of genes among bacteria within the same species. For instance, in a set of 3 genomes of *Escherichia coli* studied by Welch et al. 2002 [1] only 40% of all genes were common to all 3 genomes, and 21% of genes in the uropathogenic isolate did not exist in the other two isolates (a non-pathogenic and a enterohaemorrhagic strain). Horizontal gene transfer plays a major role in the evolution of bacterial genomes [2-4], and is one factor explaining the large extent of variation in gene presence and absence among sets of related genomes [1]. Indeed, bacterial genomes can acquire entire genetic pathways encoding discrete functions through horizontal gene transfer [5]. A major impact of this dynamic is that bacteria can acquire new functions without having to evolve all of the elements of the pathway *de novo*. When a bacterial population is faced with a new niche, this ability to acquire new pathways confers considerable advantage [2]. For example, genes for enzymes acting on polysaccharides in edible algae have been transferred from marine bacteria to human gut bacteria [6]. Many examples of the horizontal acquisition of pathogenicity islands have also been described [7-9].

The large extent of variation in gene content between related bacterial genomes suggests a corresponding variation in gene presence and absence within pathways. For example, isolates of *Neisseria meningitidis* lacking components of virulence systems have been observed [10]; variation in the presence and absence of genes involved in oxidative sulfur metabolism occurs across species of green sulfur bacteria (Chlorobium and related genera) [11]; and metagenomic analysis of human gut microflora suggests a patchy distribution of porphyranase genes of marine origin in gut bacteria of genus Bacteroides [6]. Given such observations, extensive variation in gene content within pathways is likely to be uncovered as more bacterial genomes are analysed.

A challenge is to explain variation in gene content, particularly at the pathway level. Why would there be any variation if a pathway is beneficial? One possibility is that a change to environmental conditions creates a situation in which a pathway is no longer needed, and so existing pathways undergo decay by loss of individual genes over time. This reductive evolution occurs at the genome level in some taxa [12,13].

Another possibility arises in the situation in which the pathway is not essential for survival, and in which there is little benefit in carrying it. Under this scenario, provided there is little or no cost to carrying the genes, horizontal transfer contributes to an accumulation of variation in the presence of components of the pathway. If the environment changes to one in which the pathway confers an advantage, selection would then favour acquisition of the complete set of genes. Indeed, Hall et al. 1983 [14] proposed that phenotypically silent genes or pathways may act as reservoirs for adaptation when environments change. In this context, cryptic genes may be maintained by fluctuation in environments [15]. We later discuss how this relates to the idea of cryptic genetic *variation*. A similar but distinct alternative hypothesis is that selection for the pathway acts continuously through partial exposure to the selective environment. Variation in gene presence again would be found en route to the acquisition of the complete pathway.

In this paper, we investigate these alternative explanations for the occurrence of variation in pathway content. We do this by developing an evolutionary model of pathway acquisition and gene loss for a bacterial population undergoing horizontal gene transfer and facing partial exposure to a new environment in which the pathway is favoured. We find that while pathway variation is readily generated by decay of pathways no longer under selection, horizontal gene transfer is generally unable to produce much variation unless the rate of transfer is extremely high. We interpret specific examples of variation in gene presence in bacteria in the context of these findings.

## Methods

### The model

We model a bacterial population that, through horizontal gene transfer, is able to acquire genes of a pathway that confers a selective advantage in some special “selective” environment or niche. The source of these genes is the collection of species carrying the complete pathway living in the selective environment. We consider the recipient bacterial population but not these source species. We assume this recipient population is large enough to be described by a deterministic system, that is, ignoring the effects of genetic drift. The model tracks the proportion *x*_*i*_ of the population containing *i* genes of the *n*-gene pathway in the genome. We assume that the external populations have a substantially greater effect than the recipient population on the distribution of gene content in the pool of DNA fragments available for uptake by transformation or other processes. Pathways are diverse in structure and could be network-like or contain redundancy [16]. To focus on the dynamics of acquisition and variation we assume pathway linearity and that all genes of a pathway are equally necessary.

Let *c* be the cost of carrying each gene due to expression and maintenance. In the selective environment in which carriage of the complete pathway is advantageous, this cost is overridden by a benefit and the net benefit in that environment is *b*. We assume that individuals are to some extent exposed to the selective environment. In particular, assume that a proportion *ε*of the time is spent in that environment. The fitness of an individual with *i* genes of the pathway is thus

(1) $$\;w_{i}=\left\{\begin{array}{cc} {\left(1-c\right)}^{i}\;\;\;\;\; & i=0,1,\ldots ,n-1\\ (1-\varepsilon ){\left(1-c\right)}^{i}+\varepsilon (1+b)\;\;\; & i=\mathrm{n.} \end{array}\right)$$

Genes of the pathway can be inactivated through mutation. We assume these mutations are irreversible and have in mind processes such as deletion, either of the whole gene or enough of the gene to render it unable to function in the pathway. This deletion occurs at rate *δ* per individual per generation. We assume that *δ*is significantly smaller than 1/*n*, so that we approximate the probability of one of *i* genes being deleted by *iδ*. At most one gene is deleted at a time.

We model the horizontal transfer of sets of genes belonging to the pathway as follows. Let the parameter *γ*_1_ be the probability of acquisition of a single gene in a generation. Genes that are grouped closely — that is, clustered — on the chromosome are more likely to be transferred than unclustered genes. This assumption follows the observation that the probability of transfer decreases with the length of the segment [5,17,18]. Letting each additional gene on a fragment reduce the probability of transfer by a factor of *κ*, the transfer probability for *i* genes is *γ*_*i*_ = *γ*_1_*κ*^*i*−1^ per recipient per generation. The parameter *κ* represents the degree of clustering of the genes: when *κ* is high the genes are more clustered and *γ*_*i*_ is flatter with respect to *i*. Specifically, a value of *κ* = 1 corresponds to the genes being colocated, while *κ*approaches 0 for evenly spaced genes as the genome length increases (under a model of clustering such as that of Ballouz *et al.*[18]).

Changes in *x*_*i*_ due to transfer are handled as follows. Consider increases through transfers such that individuals with *j* genes (where *j* < *i*) are converted to individuals with *i* genes through transfer of *i* − *j*,*i* − *j* + 1,…,*i*genes, where there may be some overlap with genes already present in the recipient genome. The number of such sets of genes whose transfer fits this requirement is $\overset{j}{\underset{k=0}{\sum }}\left(\begin{array}{c} n-j\\ i-j \end{array}\;\right)\left(\begin{array}{c} \;j\\ k \end{array}\;\right)$ where $\left(\begin{array}{c} \;j\\ k \end{array}\;\right)$ is the number of ways the set may overlap with the extant *j* genes, and $\left(\begin{array}{c} n-j\\ i-j \end{array}\;\right)$ is the number of ways of choosing the *i* − *j* “new” genes. To obtain the probability that a chosen set of *i* − *j* + *k*transferred genes satisfies this requirement, we divide by the number of possible choices of sets to transfer. Assuming the genes on the pathway in the external source population to be randomly arranged, this is the number of non-empty subsets of *n* genes, namely 2^*n*^ − 1. This is together multiplied by the transfer probability *γ*_*i* − *k*_. Summing over set sizes *j* results in the addition, then, of

$$\begin{array}{lll} \frac{1}{2^{n}\;-\;1}\overset{i-1}{\underset{j=0}{\sum }}\overset{j}{\underset{k=0}{\sum }}\left(\begin{array}{c} n\;-\;j\\ i\;-\;j \end{array}\;\right)\left(\begin{array}{c} \;j\\ k \end{array}\;\right)\gamma_{i-k}x_{j} & =\frac{1}{2^{n}\;-\;1}\; & \;\\ & \times \underset{0\leq k\leq j<i}{\sum }\left(\begin{array}{c} n\;-\;j\\ i\;-\;j \end{array}\;\right)\left(\begin{array}{c} \;j\\ k \end{array}\;\right)\gamma_{i-k}x_{j}\; & \; \end{array}$$

to the population carrying *i* genes from the pathway.

We note that the assumption of random arrangement of genes is not entirely realistic because local synteny would persist to some extent in the source population; however, some rearrangement weakens this synteny. Furthermore, most transfer events involve a single gene since shorter fragments are more likely to be transferred, and in these cases, synteny does not affect the dynamics. We therefore adopt random arrangement as a simplifying assumption; there may be benefit in re-examining this in future work.

Now consider decreases in *x*_*i*_ through transfer, converting individuals with *i* genes into individuals with more than *i* genes. This is given by computing the number of ways of adding *j* genes to a genome with *i* genes that changes the total number of genes from the pathway that are on the genome. For each *j* there are $\left(\begin{array}{c} n\\ \;j \end{array}\;\right)$ choices of *j* genes, and the number of these selections that are contained within the *i* we already have is $\left(\begin{array}{c} i\\ j \end{array}\;\right)$. Note that $\left(\begin{array}{c} i\\ j \end{array}\;\right)$iszero if *i* < *j*. To obtain a probability we again need to divide by the total number of non-empty subsets of *n*. Thus, summing over all possible *j*, we need to subtract

$$\frac{1}{2^{n}-1}\overset{n}{\underset{j=1}{\sum }}\left(\left(\begin{array}{c} n\\ \;j \end{array}\;\right)-\left(\begin{array}{c} i\\ j \end{array}\;\right)\right)\tau_{j}x_{i}.$$

**Figure 1:**
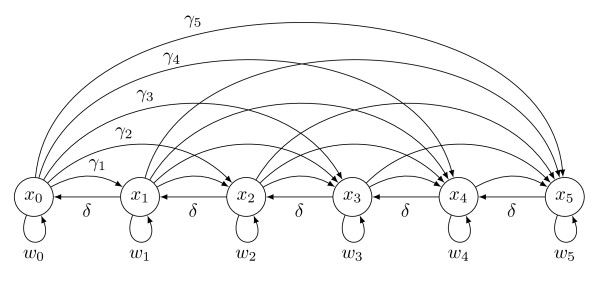
**Structure of the model.** Structure of the model for the case *n* = 5. Variables *x*_*i*_in the circles represent the proportion of the population carrying *i* genes of the 5-gene pathway in a genome. Horizontal arrows to the left indicate the effects of deletions. Curved arrows to the right show the effect of horizontal transfer. Fitnesses are depicted by arrows below populations.

Putting together all of these components and setting *x*_*n* + 1_ = 0, we have for 0 ≤ *i* ≤ *n*, the following equation describing the state of the population in the next generation (denoted by a prime).

(2) $$\begin{array}{lll} \bar{w}x_{i}^{\prime }= & \;\left(w_{i}-\mathrm{i\delta}\right)x_{i}+(i+1)\delta x_{i+1}\; & \;\\ & +\frac{1}{2^{n}-1}\underset{0\leq k\leq j<i}{\sum }\left(\begin{array}{c} n-j\\ i-j \end{array}\right)\left(\begin{array}{c} \;j\\ k \end{array}\;\right)\gamma_{i-k}x_{j}\; & \;\\ & -\frac{1}{2^{n}-1}\overset{n}{\underset{j=1}{\sum }}\left(\left(\begin{array}{c} n\\ \;j \end{array}\;\right)-\left(\begin{array}{c} i\\ j \end{array}\;\right)\right)\gamma_{j}x_{i}\; \end{array}$$

where $\bar{w}$ is a normalizing factor. It can be shown that this normalizer is the mean fitness ( $\bar{w}=\overset{n}{\underset{i=0}{\sum }}w_{i}x_{i}$). Figure 1 illustrates the structure of the model for the special case of *n* = 5.

Note that for the values of parameters we consider in this model (Table 1), which follow the constraints that *δ* ≪ 1/*n*,  *γ*_1_ ≪ 1 and *c* ≪ 1, all coefficients in the expansion of $\bar{w}x_{i}^{\prime }$ in Equation (2) are non-negative, since:

$$w_{i}\gg \mathrm{i\delta}+\frac{1}{2^{n}-1}\overset{n}{\underset{j=1}{\sum }}\left(\left(\begin{array}{c} n\\ \;j \end{array}\;\right)-\left(\begin{array}{c} i\\ j \end{array}\;\right)\right)\gamma_{j}.$$

**Table 1:** Parameters of the model

**Symbol**	**Range or value**	**Meaning**
*n*	[1, 30]	the number of genes in the pathway
*c*	[0, 0.01]	the fitness cost per gene to a single genome
*ε*	[0, 1]	the exposure probability (of encountering the selective environment)
*b*	0.1	the fitness benefit conferred by the whole pathway
*δ*	10^−9^	the probability of deletion of a single gene per generation
*γ*_1_	[10^−9^, 10^−3^]	the transfer probability for 1 gene per generation
*κ*	[0.1, 1]	the transfer decay parameter, modelling clustering

We consider properties of the model in the Results, and also analyse the dynamics numerically by iterating Equation (2) with initial conditions of either *x*_0_=1 (all individuals lack all genes of the pathway) or *x*_*n*_ = 1(all individuals have the complete pathway). We will refer to the state of lacking all genes of the pathway as the “empty pathway”. This computational work was done in the programming language Sage [19].

The parameters are set according to the default values shown in Table 1 and varied within the ranges given to explore their impact on the dynamics. We fix the length of the pathway at *n* = 5genes. Deleterious mutation occurs in *E. coli* at an estimated minimum rate of 2×10^−4^per genome per generation [20]. If there are 4000 genes, this corresponds to 5×10^−8^ per gene per generation. We set the per gene loss rate to 10^−9^, given also that not all deleterious mutations render genes non-functional. Rates of horizontal gene transfer would vary widely depending on what mechanisms of genetic exchange are operating, the availability of foreign DNA, and presumably many other factors. We therefore consider two rates: a low rate of the same order of magnitude as the rate of gene loss, namely 10^−9^, and a high rate of 10^−3^ corresponding to the presence of highly mobile plasmids.

The metabolic cost of carrying genes is likely to be low [21], so we consider values in the range 0 to 0.01. The fitness *w*_*n*_ of carrying the full pathway ranges from (1 − *c*)^*i*^when *ε* = 0 to 1 + *b* when *ε* = 1, and so the parameter *b* sets an upper limit for the overall fitness conferred by possession of the full pathway. We fix this at *b* = 0.1, and allow *ε* to vary in the range [0,1].

## Results

### The equilibrium

This system has a unique equilibrium, as the following shows. The state transitions of this model can be described by the equation $\bar{w}x^{\prime }=Ax$ where **x** is a state vector **x** = (*x*_0_,*x*_1_,…,*x*_*n*_)^*T*^, and *A* is the matrix whose (*i*,*j*) entry is given by the coefficient of *x*_*j*_ in the expansion of $\bar{w}x_{i}^{\prime }$ in Equation (2). This is a *non-linear* system, since $\bar{w}$, which is also the mean fitness of the population, depends on the state **x**.

Despite the system’s non-linearity, we can obtain the existence and uniqueness of an equilibrium state through considering the matrix *A* in the light of the *Perron-Frobenius Theorem* for irreducible matrices (see, for example, [22, Section 8.3]). This Theorem states that *a non-negative irreducible matrix has a unique eigenvector that is a scalar multiple of a positive vector*. The associated (positive) eigenvalue is the spectral radius of the matrix and is called the Perron root, and the positive eigenvector is called the Perron vector.

The (*n* + 1)×(*n* + 1)matrix *A* that arises in our model is irreducible because it is regular, meaning that a positive power of *A* has strictly positive entries. This in turn follows because the entries of *A* are all strictly positive on and below the main diagonal, as well as in the first diagonal above the main diagonal (the remaining entries are zero). Hence *A*^*n*^ will have only positive entries, implying that *A* is regular. The Perron-Frobenius Theorem therefore applies, and so *A* has a unique eigenvector that is a scalar multiple of a positive vector. In other words, there is an eigenvector of *A* that consists of positive entries, and with the additional constraint that the entries sum to 1 this vector (that is, state) is unique. In concrete terms, there is a unique positive vector **v** summing to 1 such that *A***v** = *λ***v**, with *λ* > 0. This provides a unique solution to our system’s equilibrium equation $Ax=\bar{w}x$, namely **x** is the Perron vector **v** and $\bar{w}$ is the Perron root *λ*.

An additional consequence of the regularity of *A* is that the dynamics are pushed away from the boundaries of the positive cone of the state space. In other words, there is *protected polymorphism*. Specifically, once the state vector is strictly positive, which occurs after at most *n* steps, no variable *x*_*i*_ can ever again be zero because the transition matrix has non-negative entries.

A consideration of the fitnesses reveals conditions under which the population is closer to being either fixed on the complete pathway (*x*_*n*_ high) or fixed on the empty pathway (*x*_0_ high). The cost *c* favours the empty state while exposure *ε*favours the full pathway, and the balance between these two factors is seen by comparing the fitnesses of the two extreme states. The separation between high *x*_0_and high *x*_*n*_ should occur when *w*_*n*_=1=*w*_0_which holds when

(3) $$\varepsilon =\varepsilon^{*}=\frac{1-{\left(1-c\right)}^{n}}{1+b-{\left(1-c\right)}^{n}},$$

following from the definition of *w*_*i*_ (Equation (1)). That is, under this heuristic, when *ε* > *ε*^∗^ the dynamics should move to high values of *x*_*n*_at equilibrium. Note that for a given fitness cost *c*, as the pathway length *n* increases the exposure *ε* required in order for the pathway to become fixed in the population also increases.

### Dynamics

We study the transient dynamics of pathway acquisition numerically by setting the parameters according to Table 1 and iterating the process (Equation (2)) until the variables are within a small tolerance of equilibrium. For each set of parameters, the dynamics were examined from two boundary state initial conditions: 1. no individuals in the population carried any genes in the pathway (fixation on the empty pathway, *x*_0_=1), and 2. All individuals in the population carried all of the pathway (fixation on the complete pathway, *x*_*n*_=1). We use these initial conditions to understand how genes of a pathway are acquired and lost; other initial conditions would also lead to the unique internal equilibrium point described above.

**Figure 2:**
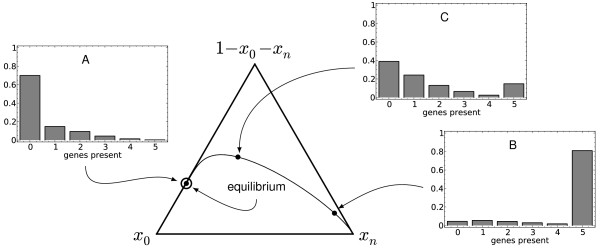
**Example de Finetti diagram.** Example of a de Finetti representation of the trajectory of the process with 5 genes, with parameters set at *γ*_1_ = 10^−3^, *κ* = 0.5, *c* = 0.0008and *ε* = 0.003. These parameters reflect a high rate of transfer, and low exposure to the selective environment. The central figure shows the change in population from each end, with the equilibrium state shown by a circle on the left hand side. Histogram **A** shows the population distribution at equilibrium, while Histograms **B** and **C** show states along the decay trajectory from the complete pathway.

A convenient way to represent the trajectory of the population through (*n* + 1)-dimensional space is by compressing some coordinates and using a *de Finetti* diagram (see for instance [23]) as follows. A distribution of population proportions is given by a point in the interior of an equilateral triangle whose corners represent *x*_0_, *x*_*n*_ and 1−*x*_0_−*x*_*n*_. The value of each of these three variables is given by the perpendicular distance from the side opposite the vertex labelled by the variable. The quantity

$$1-x_{0}-x_{n}=\overset{n-1}{\underset{i=1}{\sum }}x_{i}$$

gives the proportion of the population with at least one of the genes, but not the complete pathway, and this reflects the extent of variation in gene content in the pathway. Figure 2 illustrates an example of a de Finetti diagram together with population distributions along the trajectory.

To study how the dynamics of this system are affected by horizontal transfer and natural selection, we varied parameters controlling these features of the model (see Table 1). Transfer is controlled by the base rate *γ*_1_ of transfer of a single gene and the extent of clustering *κ*, while selection is controlled by *c*, *b* and *ε*.

**Figure 3:**
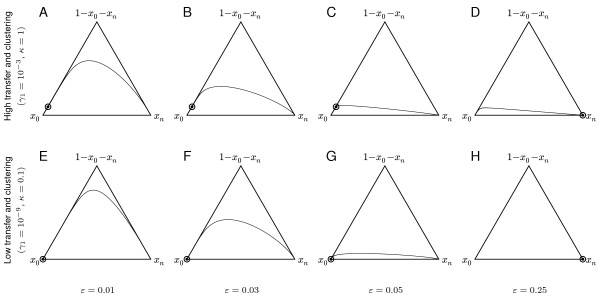
**Dynamics on de Finetti coordinates.** Dynamics starting from fixation on empty pathways and complete pathways shown on de Finetti co-ordinates. Top row (panels **A**–**D**): high transfer means *γ*_1_ = 10^−3^and *κ* = 1.0; bottom row (**E**–**H**): low transfer means *γ*_1_ = 10^−9^and *κ* = 0.1. The other parameters are *c* = 0.005,*b* = 0.1,*n* = 5,*δ* = 10^−9^.

Figure 3 shows how varying exposure *ε*for two different values of transfer rate *γ*_1_ influences the dynamics. As exposure increases, the equilibrium shifts from near fixation on the empty pathway to near fixation on the complete pathway. For the parameters used in this figure, the threshold exposure is *ε*^∗^ = 0.198 which lies between the third and fourth columns. Also, the trajectories become flatter (less variation) as exposure increases. The overall appearance of the dynamics is similar for high and low transfer settings, even though the base transfer rates differ by six orders of magnitude. The equilibrium is more polymorphic in the case of the high transfer rate.

**Figure 4:**
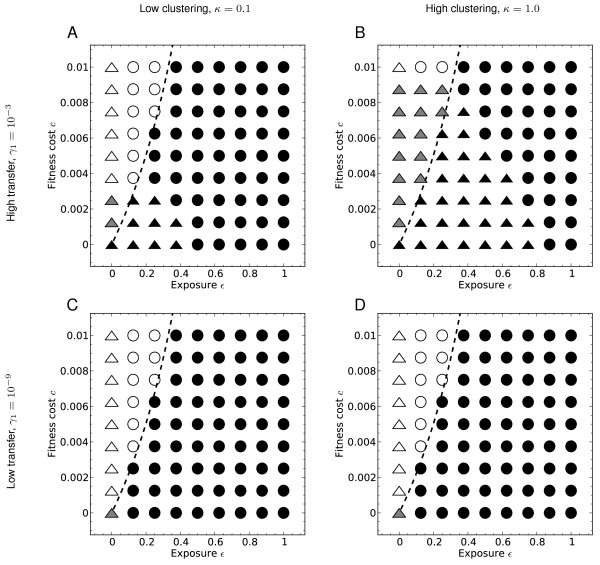
**Dynamics under different transfer scenarios.** Features of the dynamics in different transfer and clustering scenarios, as fitness cost *c* and exposure to the beneficial environment *ε*are varied. The equilibrium position is shown by the shading at the relevant point in parameter space. If the equilibrium state has *x*_*n*_ > 0.95, meaning that more than 95% of the population carries the complete pathway, the parameter space point is shown shaded in black. If instead *x*_0_ > 0.95, the shape is unshaded. If the equilibrium is more than 0.05 from each extreme, the shading is grey. A circle at a point indicates that the peak of the trajectory is below 0.05, while a triangle indicates that the peak is above 0.05. The dashed curve shows the boundary given by Equation (3). The other parameters are *b* = 0.1,*n* = 5,*δ* = 10^−9^.

We further characterised transfer and selection as follows. As before we examine low and high transfer rates (*γ*_1_), and low and high clustering (modelled by *κ*), but consider all four scenarios (Figure 4). For each of these we explored the dynamics of the process varying fitness cost *c* and exposure *ε*(recall the value of *b* is fixed at 0.1). We allowed *c* to vary between 0 and 0.01, and *ε* to vary between 0 and 1, dividing each parameter range into nine values (eight non-zero). The output quantities of interest here are the *equilibrium point* and the *extent of variation* in pathway content. The equilibrium point tells us whether the full pathway or the empty pathway is fixed or nearly fixed in the population, or if there is notable variation in the population at equilibrium. The extent of variation in gene content generated in the trajectory is captured by the peak variation (1 − *x*_0_ − *x*_*n*_) during the dynamics.

### No cost and no benefit

The first question to ask of this model is how it behaves in the absence of any cost to carrying a partial pathway and no benefit from the acquisition of the full pathway. In this way the impact of the transfer dynamics is isolated. Since we have fixed the benefit *b*=0.1, the lack of benefit is modelled by setting exposure to zero, and the cost *c* of carrying a gene is also set to zero. This situation corresponds to the bottom left hand point of each panel in Figure 4. The behaviour of the model depends on the value of *γ*_1_. If the transfer rate is high (panels A and B), the full pathway is acquired by over 95% of the population (indicated by a black shape). If the transfer rate is low (panels C and D), we do not have 95% of the equilibrium population at either the full or empty pathway (indicated by a greyed shape).

These results reflect the balance between the only forces in play without fitness differences: transfer (pushing towards the pathway) and deletion (pushing away). The deletion rate is fixed at 10^−9^, and so when the transfer rate is of a similar order of magnitude *γ*_1_ = 10^−9^(regardless of *κ*), as in the lower panels, equilibrium is near neither “corner” (*x*_0_ = 1or *x*_*n*_ = 1). In the case of a higher transfer rate (*γ*_1_ = 10^−3^, upper panels, Figure 4) deletion is overpowered and the pathway is acquired.

### Pathway acquisition

The predicted threshold given by Equation (3), shown as a dashed curve on Figure 4, proves to be accurate: instances of fixation or near fixation on complete pathways (represented by black shapes) lie on the right of the dashed curve. Here, the exposure to the selective environment is high enough compared to the cost to allow pathway acquisition. To the left of the dashed curve, where exposure is low compared to cost, are instances of empty pathways (white shapes) or polymorphic equilibria (grey shapes). The latter outcomes, in which the equilibrium is neither close to the complete nor to the empty pathway, indicate a stable state for which there is substantial variation in gene presence. These only occur when exposure to the selective environment is very low, and the fitness cost of carrying a gene is also low or zero, with one exception: when the horizontal transfer parameters favour frequent transfer and high gene clustering (Figure 4B, high *γ*_1_ and high *κ*). In this case, variation at equilibrium appears in a wider range of parameter values.

### Variation in gene presence

As mentioned above, variation in the presence of genes from a pathway means that a significant proportion of the population contains neither the complete pathway nor the empty pathway. In our context, we define this be when at least 5% of the population are carrying neither of these extremes. The maximum value of 1 − *x*_0_ − *x*_*n*_ is an indicator of this variation. On the de Finetti diagram of a given trajectory (e.g. see Figures 2 and 3), this corresponds to the maximum height attained by the trajectory. Such variation can arise in several ways.

Firstly, at equilibrium as noted above, variation in pathway content can occur when the exposure *ε* is sufficiently low compared to cost *c* and the transfer rate *γ*_1_ is high (Figure 3A–C and grey triangles in Figure 4A and B). This high level of variation occurs because the force of horizontal transfer is strong enough to counter the loss of genes.

Variation can also arise in *transition* towards equilibrium, either from the empty or the complete pathway. In the case of the trajectory en route to acquisition, observe that genes are transferred in the model in groups of any number but with decreasing rates for larger numbers. Thus, depending on the balance of parameters, the pathway may be acquired directly by transfers of large numbers of genes, or more gradually with small numbers of genes at a time. Variation en route to acquisition is represented by black triangles in Figure 4, and occurs when the transfer rate is very high, and is more pronounced when clustering (represented by *κ*) is also high (Figure 4B and Figure 3D). In particular, from Figures 4A and 4B we see that variation en route to acquisition occurs when both cost *c* and exposure *ε*are low.

Variation can also arise in transition in the other direction, through the loss of genes starting from a complete pathway. Indeed, when transfer rates are low, the only significant variation in the presence of genes from the pathway arises when gene loss occurs starting from the complete pathway (Figures 4C and D and Figures 3E–G). This occurs when exposure to the selective environment is very low. The same phenomenon arises for high transfer: absence of exposure to the selective environment results in slow decay that passes through significant variation.

This evolutionary passage of decay through intermediate numbers of genes occurs when the fitness benefit of acquiring the full pathway does not outweigh the additional cost of the final gene. In this situation, there is no contest between the complete pathway and the empty pathway, unlike the balance leading to the threshold of Equation (3). A second threshold exposure derives from this argument, namely at *w*_*n*−1_ = *w*_*n*_, or

(4) $$\varepsilon^{**}=\frac{c{\left(1-c\right)}^{n-1}}{1+b-{\left(1-c\right)}^{n}}\leq \varepsilon^{*}.$$

Roughly speaking, for exposure levels lower than this, large amounts of variation in gene presence appear as pathways decay. When exposure levels are higher than *ε*^∗∗^ but still lower than *ε*^∗^, selection leads to pathway decay but through a more direct replacement with the empty pathway. The threshold given in Equation (4) corresponds to a very low level of exposure, closer to the far left column of the panels in Figure 4 than to the second column (representing *ε*=0.125). Figure 3 illustrates how de Finetti trajectories change around this threshold, with a value of *ε*^∗∗^ = 0.039 for these parameters lying between the second and third columns.

When there is no exposure to the selective environment at all (*ε* = 0) — the left columns of each panel in Figure 4 — we see the greatest extent of variation (represented by triangles). Most such variation arises from pathway decay, but when the transfer rate is high enough, as in panels A and B of Figure 4, polymorphic equilibria (grey triangles) indicate that variation has accumulated from the empty pathway in the absence of selection. Even in this situation, when the cost is too high little variation accumulates (white triangles at the top of the left axes in panels A and B) reflecting the dynamic balance between transfer and fitness cost.

Flat trajectories that do not reach 0.05 in height – so that 95% of the population mass remains with *x*_0_ and *x*_*n*_ at all times – tend to appear when exposure is high (circles in Figure 4). These points are where the pathway is acquired through transfers of large numbers of genes. For low transfer rates (Figure 4C and D), this is the only way that pathways are acquired. An example of such a trajectory is shown in Figure 3H. When there is a higher rate of transfer (Figure 4A and B), this outcome occurs less readily, especially when clustering is high (Figure 4B). The inverse outcome, in which the pathway is acquired in pieces (black triangles in Figure 4), is only seen when the transfer rate is high, and in particular when the degree of gene clustering *κ* is also high (Figure 4B). Figure 3D shows this scenario on a de Finetti diagram. Even here, however, the trajectory is relatively flat, indicating only a low level of transient variation. Note that gene clustering *κ*increases the transient peak variation, but only when the transfer rate *γ*_1_ is high.

### Effect of pathway length

We varied the length of the pathway to study the effect of this parameter on the model behaviour. To cover realistic pathway lengths we explored a wide range of values (*n* = 1,…,30). In the extreme case, when *n* = 1, there are only two states in the system, presence or absence of the pathway. Consequently there is no “variation” in gene presence in the sense of there being partial pathways. However the *equilibrium* behaviour can be established. Because there are only two coupled states, the system reduces to a quadratic in one variable, namely

$$(1-w_{1})x_{1}^{2}-(1-w_{1}+\gamma_{1}+\delta )x_{1}+\gamma_{1}=0.$$

In the light of the discussion of the equilibrium above, exactly one of these gives a positive solution for *x*_0_ and *x*_1_ under realistic parameter choices. In the case that there is no fitness cost and no exposure to the beneficial environment (*c* = *ε* = 0), we have both states equally fit (*w*_0_ = *w*_1_ = 1), and the system becomes linear. The dynamics are then a balance between transfer *γ*_1_ and deletion *δ*, and the equilibrium is simply *x*_0_ = *δ*/(*δ* + *γ*_1_) and *x*_1_ = *γ*_1_/(*δ* + *γ*_1_). If deletion and transfer occur at the same rate, the equilibrium is (*x*_0_,*x*_1_) = (1/2,1/2).

**Figure 5:**
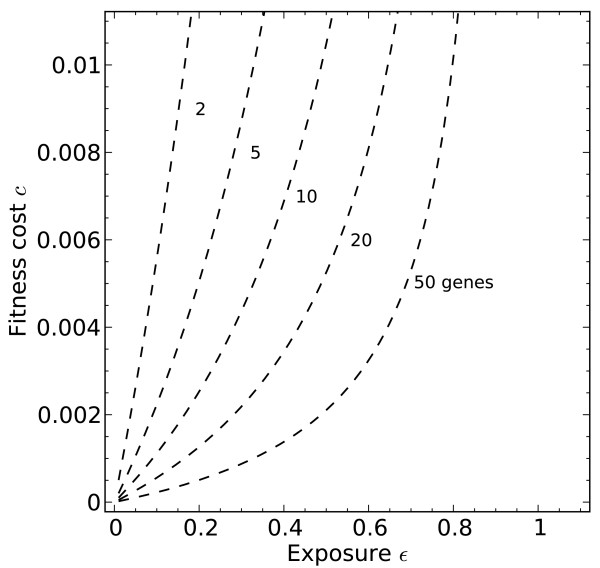
**The pathway acquisition threshold*ε***^**∗**^(Equation (3)) **shown as pathway length*n*varies.** This heuristic threshold relationship between *c* and *ε*is dependent only on *n* and the fitness benefit *b*, which is set to *b* = 0.1.

**Figure 6:**
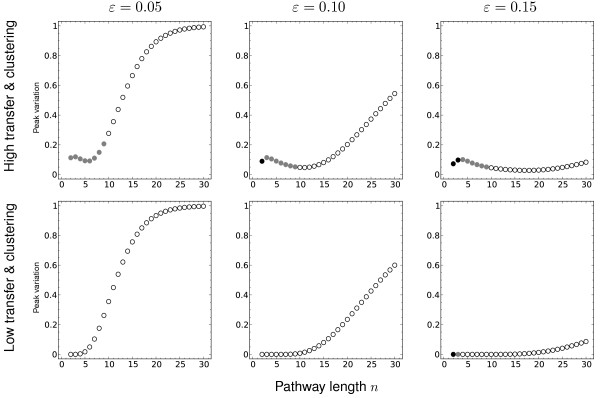
**Variation in gene presence for different pathway length.** Changes in peak variation in gene presence shown as a function of the pathway length *n*. Each data point shows the maximum value of 1 − *x*_0_−*x*_*n*_attained in the course of the numerical process, for the given parameter values. The shading at each point indicates the position of the equilibrium state: black indicates pathway acquired (*x*_*n*_>0.95), white indicates pathway absent (*x*_0_>0.95), and grey indicates the equilibrium is more than 0.05 from each extreme. The transfer rates used were *γ*_1_ = 10^−3^(high) and 10^−9^(low), and clustering *κ* = 1.0(high) and 0.1 (low). Throughout, fitness cost *c*, fitness benefit *b* and deletion rate *δ*are fixed at *c* = 0.005, *b* = 0.1and *δ* = 10^−9^.

For *n*>1 pathway acquisition requires more exposure to the beneficial environment, relative to fitness cost. That is, the pathway acquisition threshold *ε*^∗^(Equation (3)) moves to the right as shown in Figure 5. The effect of pathway length on variation in gene presence is more subtle. Figure 6 shows the effect of increasing length while keeping cost *c* fixed but varying exposure *ε* in both high and low transfer scenarios. The immediate observation is that variation is more common with longer pathways, as might be expected. It is also clear that this variation occurs in the course of pathway loss (indicated by unfilled circles in Figure 6), rather than acquisition, consistent with our observations for 5-gene pathways. There are some differences between low and high transfer rate scenarios (top vs bottom row of Figure 6), chiefly, variation at low *n* and acquisition behaviour as *ε*increases. With low *n* and high transfer (top row, Figure 6), the significant difference between the transfer rate *γ*_1_ and the deletion rate 10^−9^gives rise to variation en route to an equilibrium with the full pathway or in between. The slight difference in acquisition behaviour between high and low transfer rates over various *n* concurs with the difference seen in Figure 4B and C along the dotted line heuristic threshold.

## Discussion

Analysis of the model shows that with continuous partial exposure to the selective environment a pathway can be acquired when the level of exposure is high enough compared to the cost of carrying fragments of the pathway. Continuous partial exposure may explain, for instance, the acquisition of porphyranase genes from marine bacteria by human gut bacteria. From the perspective of the gut microbes, the diet of humans ingesting seaweed provides constant exposure to the selective environment containing polysaccharides that marine bacteria are able to metabolise. Because genes of a pathway are often clustered on chromosomes [5,24], horizontal transfer can move multiple genes, which accelerates the process of acquisition. Indeed, a bioinformatic analysis of pathways shows that acquisition is mostly rapid rather than gradual [25]. The speed of acquisition clearly depends on the extent of exposure to the selective environment.

We have shown that some variation in the presence of genes of the pathway can accumulate when the selective environment is absent, or nearly absent, in certain conditions. Such variation is seen in Figure 4 where variation is evident along the left axis of each panel (as well as in Figure 6 when the pathway is long and exposure very low). This scenario is very similar to the notion of *cryptic genetic variation*, or evolutionary capacitance, which is usually discussed in the context of eukaryotes [26-28]. In principle cryptic genetic variation can also apply to prokaryotes. We contrast this concept with *cryptic genes* in the sense of Hall et al. 1983 [14] who have in mind complete rather than partial pathways, which are maintained by alternating environments. Here, we are interested in variation: pathways can be built from scratch through horizontal transfer, and swiftly be driven to fixation when the appropriate environment arises. Hence, this process is much like cryptic genetic variation, except that the mechanism for variation accumulation is horizontal transfer rather than mutation. Based on our results, however, this kind of variation only occurs when the rate of horizontal transfer is very high and pathway genes are clustered (Figure 4).

Various genetic structures and mechanisms promote the transfer of genes between bacterial genomes. Integrons, for example, capture genes in cassette structures and are notable for their role, in conjunction with plasmids, in the accumulation of multiple drug resistance genes in pathogens [29,30]. Here, horizontal transfer plays a far greater role in generating variation for selection to act on than does mutation [31]. When mobile genetic elements harbour genes that arose by transfer rather than being directly selected, this is very much like cryptic genetic variation. Although multiple drug resistance is not an instance of a metabolic pathway it falls into the scope of this study: multiple genes are required to generate a fitness advantage in special environments in which many drugs are found. Such an environment may be an infected host who is treated either sequentially or simultaneously with multiple antibiotics. If continuous partial exposure to this environment contributes to the maintenance of the multiple resistance phenotype, then the variation is not so cryptic and these genes are more like cryptic genes in Hall’s sense [14].

Our model shows how variation in gene presence can also occur through decay of an existing pathway. This type of variation occurs when exposure is very low or zero; that is, when the selective environment no longer acts on the pathway. Here, the pathway is mostly lost at equilibrium but there is a large degree of variation produced transiently as the pathway decays. This variation in gene content is generally far greater than the variation due to the accumulation of genes by horizontal transfer (Figure 3). The model structure (see Figure 1) shows why this is so. The process of acquisition by horizontal transfer can be a rapid process as described above because multiple genes can be gained at once. In contrast, deletion of genes occurs gradually – one gene at a time. While acquisition occurs without much intermediate variation, decay from a complete pathway takes a different route, visiting these intermediate states. Thus, where large amounts of variation occur in pathways, such observations would be better explained by past changes in the environment so that the genes in question were no longer being needed or were only weakly selected because of genetic drift [32]. The extensive genome variation due to gene decay observed in *Shigella*[13,33] and *Mycobacterium leprae*[12] is a clear illustration of this point.

Horizontal gene transfer has frequently been argued to be an important force in bacterial genome evolution [2]. This stems from the observation of frequent transfer events that cross prokaryotic species boundaries and the inevitable distortion of phylogenetic signal that results [34]. This idea is strengthened by the observations of the transfer of whole pathways across species and the mixed origins of elements of pathways [35,36]. Our study examines the impact of horizontal gene transfer as an evolutionary force acting within a bacterial species, especially on the extent of variation at the pathway level. Although horizontal gene transfer is likely to play a large role in the initial acquisition of pathways, it is far less able to explain pathway variation than can mutational loss of genes.

As more genomic data become available, a careful examination of variation within pathways within species will shed light on these ideas. If the distribution of the number of genes in a pathway is either weighted towards the complete pathway or weighted in the middle (e.g., Figure 2B or 2C), then it is likely to be a decaying pathway in transit. If on the other hand, the distribution is weighted towards zero elements of the pathway, then this distribution could be consistent with either a small accumulation of variation due to horizontal transfer, or the decay of an existing pathway in its final stages (e.g. Figure 2A). In this case an examination of the phylogenies of the remaining genes would allow the issue to be resolved. If those trees are congruent (and follow the tree of vertical evolution), then the observed pattern arose by decay provided there have not been subsequent transfer events to swamp the signal; otherwise it was from horizontal transfer. Understanding variation in presence and absence of genes in pathways can provide insight into the current ecology of a species as well as its evolutionary history.

## Conclusion

Analysis of the mathematical model developed in this study has shown that a high level of variation in gene presence in bacterial genomes can be readily explained through decay of an existing pathway. In the context of pathway variation the role of horizontal gene transfer is probably the initial introduction of a complete novel pathway rather than in building up the variation in a genome without the pathway.

## Authors’ contributions

ARF and MMT designed the study, performed the research, and wrote the manuscript. Both authors read and approved the final manuscript.
